# Endosymbionts moderate constrained sex allocation in a haplodiploid thrips species in a temperature-sensitive way

**DOI:** 10.1038/s41437-022-00505-5

**Published:** 2022-02-03

**Authors:** Alihan Katlav, Duong T. Nguyen, Jennifer L. Morrow, Robert N. Spooner-Hart, Markus Riegler

**Affiliations:** 1 Hawkesbury Institute for the Environment, Western Sydney University, Locked Bag 1797, Penrith, NSW 2751 Australia; 2 New South Wales Department of Primary Industries, Elizabeth Macarthur Agricultural Institute, Private Bag 4008, Narellan, NSW 2567 Australia

**Keywords:** Evolutionary genetics, Evolutionary ecology, Parasitology

## Abstract

Maternally inherited bacterial endosymbionts that affect host fitness are common in nature. Some endosymbionts colonise host populations by reproductive manipulations (such as cytoplasmic incompatibility; CI) that increase the reproductive fitness of infected over uninfected females. Theory predicts that CI-inducing endosymbionts in haplodiploid hosts may also influence sex allocation, including in compatible crosses, however, empirical evidence for this is scarce. We examined the role of two common CI-inducing endosymbionts, *Cardinium* and *Wolbachia*, in the sex allocation of *Pezothrips kellyanus*, a haplodiploid thrips species with a split sex ratio. In this species, irrespective of infection status, some mated females are constrained to produce extremely male-biased broods, whereas other females produce extremely female-biased broods. We analysed brood sex ratio of females mated with males of the same infection status at two temperatures. We found that at 20 °C the frequency of constrained sex allocation in coinfected pairs was reduced by 27% when compared to uninfected pairs. However, at 25 °C the constrained sex allocation frequency increased and became similar between coinfected and uninfected pairs, resulting in more male-biased population sex ratios at the higher temperature. This temperature-dependent pattern occurred without changes in endosymbiont densities and compatibility. Our findings indicate that endosymbionts affect sex ratios of haplodiploid hosts beyond the commonly recognised reproductive manipulations by causing female-biased sex allocation in a temperature-dependent fashion. This may contribute to a higher transmission efficiency of CI-inducing endosymbionts and is consistent with previous models that predict that CI by itself is less efficient in driving endosymbiont invasions in haplodiploid hosts.

## Introduction

Inherited bacterial endosymbionts are common in nature and play pivotal roles in their hosts’ biology, ecology and evolution (Buchner 1965). Some endosymbionts are beneficial because they confer a fitness advantage on their hosts by provisioning essential nutrients or providing protection against pathogens or environmental extremes (Douglas 2015); others are more parasitic and manipulate host reproduction to facilitate their transmission in host populations (Stouthamer et al. 2002; Oliver et al. 2010; Douglas 2015). *Wolbachia* and *Cardinium* bacteria are ubiquitous endosymbionts of arthropods (Weinert et al. 2015) and are inherited via the egg cytoplasm, while they are removed from sperm cells during spermatogenesis and, therefore, their paternal inheritance is very rare (Bressac and Rousset 1993; Doremus et al. 2020). Their maternal transmission has resulted in the evolution of host reproductive manipulations that select for an increased proportion of infected females in host populations (Hurst and Frost 2015; Zug and Hammerstein 2015). Common mechanisms of host reproductive manipulations are the induction of cytoplasmic incompatibility (CI), thelytokous parthenogenesis, male killing and feminization (Stouthamer et al. 2002). Furthermore, *Wolbachia* and *Cardinium* sometimes coinfect individuals of host species, and in such situations, either one (White et al. 2009) or both (Nguyen et al. 2017) can independently cause host reproductive manipulations. While affecting reproductive systems of hosts, reproductive manipulators that manipulate sex ratios also intrinsically affect sex allocation (Werren and Beukeboom 1998; Vala et al. 2003), and manifestation of this can vary depending on the hosts’ sex determination system (Kageyama et al. 2012).

The most common form of host reproductive manipulation, CI, in its simplest form, occurs when eggs of an uninfected female are fertilized with sperm of an infected male (Werren et al. 2008). In diplodiploid species, CI leads to embryonic mortality irrespective of the embryo’s sex (Werren et al. 2008). However, this is not the case for haplodiploid species in which females can adjust production of diploid daughters and haploid sons via fertilisation control. In haplodiploids, CI can lead to either the mortality of fertilized eggs (female mortality FM-CI) or the conversion of individuals developing from fertilized eggs into males (male development MD-CI); in contrast, unfertilised eggs remain unaffected and develop into males (Bordenstein et al. 2003; Vavre et al. 2003; Ros and Breeuwer 2009).

In general, CI can confer a fitness advantage upon infected females as they are compatible with both infected and uninfected males, and, therefore, capable of offspring (including female) production, increasing the proportion of infected individuals which then increases the endosymbionts’ prevalence and invasion success (Turelli 1994). However, a high frequency of uninfected individuals in host populations can hinder a CI-driven endosymbiont invasion. In particular, in haplodiploids the production of uninfected males in FM-CI, and even more so in MD-CI, may impede endosymbiont invasion. This is mainly because these uninfected males can subsequently mate with their uninfected mothers or other uninfected females which results in the production of uninfected females (Egas et al. 2002). Therefore, compared to diplodiploid hosts, haplodiploids require a higher proportion of infected individuals (i.e., a higher infection threshold level) before a CI-driven endosymbiont invasion can occur (Egas et al. 2002).

Theory suggests that in haplodiploid hosts, endosymbionts may require complementary strategies to increase the efficiency of CI. One strategy may be the manipulation of the host’s sex allocation towards more female production, including by compatible infected pairs, and there is some evidence for this in the *Wolbachia*-infected spider mite *Tetranychus urticae* (Vala et al. 2003) and two *Wolbachia*-infected parasitoid wasp species (Vavre et al. 2000; Bordenstein and Werren 2000). Sex allocation influenced by other endosymbionts, *Hamiltonella* and *Rickettsia*, has also been documented in a whitefly species (Shan et al. 2019; Himler et al. 2011) and, for *Wolbachia*, even in a diplodiploid sheetweb spider (Gunnarsson et al. 2009). Nevertheless, the generality of this endosymbiont effect in other haplodiploid taxa and their underlying mechanisms remain unknown. Furthermore, how important abiotic factors such as temperature may affect endosymbiont-induced sex ratio biases is understudied, despite substantial evidence of the effects of temperature on endosymbiont densities and the expression of reproductive manipulations such as CI and male killing, and, consequently, endosymbiont invasion dynamics (Hurst et al. 2000; Corbin et al. 2017; Doremus et al. 2019).

Thrips (Thysanoptera) have evolved haplodiploidy independently from Hymenoptera and other haplodiploid lineages such as wasps (Hymenoptera), scales and whiteflies (Hemiptera), and mites (Acariformes) (Evans et al. 2004; Nguyen et al. 2015). As with all haplodiploids, their maternal ability of sex ratio adjustment via fertilization control in response to biotic and abiotic conditions make thrips important model organisms for testing the generality of sex allocation theories in haplodiploids (Crespi 1992; 1993; Katlav et al. 2021a). In this study, we examined the effect of bacterial endosymbionts on the sex allocation in *Pezothrips kellyanus* (Thripidae), a native Australian haplodiploid thrips species which is naturally infected with one *Cardinium* strain and one *Wolbachia* supergroup B strain (Nguyen et al. 2016). This species reproduces by arrhenothoky, as only mated females can produce female offspring (Varikou et al. 2012; Nguyen et al. 2017). Based on comprehensive crossing experiments between infected and uninfected individuals, with analyses of egg hatch and survival to adulthood, it was found that both bacterial endosymbionts independently induce CI, with their coinfection resulting in FM-CI; however, other reproductive manipulations such as male killing and feminization were excluded (Nguyen et al. 2017). Interestingly, females of *P. kellyanus* display condition-dependent constrained sex allocation which leads to split sex ratios among mated females, i.e., small mated females, despite successful insemination, produce extremely male-biased broods, whereas large mated females produce extremely female-biased broods (Katlav et al. 2021a), possibly to mitigate the costs of constrained sex allocation at the population level (Godfray 1990). However, it remains unknown whether the two maternally inherited endosymbionts can affect this split sex ratio pattern and/or whether the split sex ratio pattern is temperature-dependent. These questions are important as answers may apply to other haplodiploids more widely, including social hymenopterans in which split sex ratios are common and mainly associated with colony-based relatedness asymmetry (Keller et al. 2001).

Previous laboratory and field studies of *P. kellyanus* reported temperature-dependent and seasonally fluctuating sex ratio patterns at population levels (Varikou et al. 2012; Navarro-Campos et al. 2013); yet it was unknown whether these patterns arose from endosymbiont infections and/or temperature effects on the sex ratio of individual broods. Here, we tested whether (1) individual females of a population naturally coinfected by *Cardinium* and *Wolbachia* are less likely to experience constrained sex allocation than uninfected individuals from which the endosymbionts had been removed by antibiotic treatment, (2) temperature moderates any such endosymbiont effects on constrained sex allocation, and (3) any such temperature effects on endosymbiont-influenced sex allocation correlate with temperature-dependent changes in endosymbiont densities and/or CI strength. Addressing these questions will help further uncover the epidemiological dynamics of endosymbionts in haplodiploid host populations and reveal factors that drive the evolution of constrained sex allocation and split sex ratios.

## Methods

### Establishment and maintenance of laboratory lines

A laboratory population of *P. kellyanus* originally established with individuals collected from the Riverland, South Australia, was used for this experiment. This population was naturally coinfected (I_CW_) with one *Cardinium* strain and one *Wolbachia* supergroup B strain (Supplementary Information; Nguyen et al. 2016). Thrips were reared as described by Nguyen et al. (2017). All the experiments were conducted in environmental chambers set at controlled conditions of 70% ±1.6 relative humidity (mean ± SE), 16:8 h (light:dark) and 20 °C ± 0.04 (experiments 1–3) or 25 °C ± 0.05 (experiment 3). To establish an uninfected line (U), adults of the coinfected line (I_CW_) were treated with a 5% rifampicin (w/v) solution for three generations (Nguyen et al. 2017) followed by recovery from antibiotic effects for at least two generations prior to the experiments. Individuals were tested for endosymbiont infection status as described by Nguyen et al. (2016).

### Experimental design

**Fig. 1:**
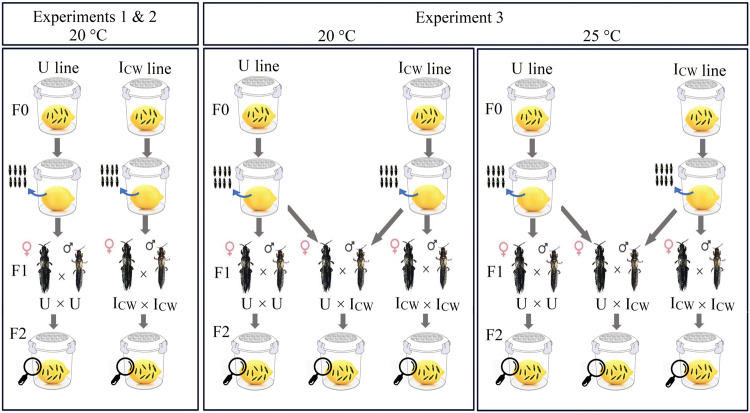
Experimental design used to assess offspring number and sex ratio of compatible pairs of *Pezothrips kellyanus* infected with *Cardinium* and *Wolbachia* (I_CW_ × I_CW_) or uninfected (U × U). Experiment 3 also included the incompatible cross (U × I_CW_). Experiments 1–2 were performed at 20 °C, while experiment 3 was performed at both 20 and 25 °C. Thrips were kept at temperature conditions for three generations (F0 to F2). F0 were kept at controlled density to produce the F1 pairs used in crossing experiments, and their offspring number and sex were assessed at the adult stage of F2. For better visualization, the thrips are depicted larger than their actual size.

Data were collected in three consecutive crossing experiments (Fig. 1) that assessed offspring number and sex ratios at the adult stage in crosses between compatible pairs of coinfected (I_CW_ × I_CW_) and uninfected (U × U) individuals; experiment 3 also included the CI cross (U × I_CW_). Singly infected lines were not available when the experiments were conducted. Therefore, experiments 1 and 2 addressed the combined effect of both endosymbionts on offspring number and sex ratios of females reared at 20 °C only, whereas in experiment 3, the offspring number and sex ratio effects of the coinfecting endosymbionts were assessed at 20 and 25 °C. At these temperatures different sex ratios have previously been reported for *P. kellyanus* at the population level (Varikou et al. 2012), and, generally, these temperatures are experienced by *P. kellyanus* in the field. The temperature treatments were implemented for at least two generations prior to the experimental data collection; i.e., F0 individuals were reared at the experimental temperatures from egg to adult, the F1 offspring were used to set up crossing experiments and their offspring (F2) number and sex ratio were measured at the adult stage. *Cardinium* and *Wolbachia* infections of each line were confirmed by PCR screening of randomly selected 20 females and 20 males per line before each experiment commenced.

For the crossing experiments, we used similar-aged individuals (1 day old virgin females and 1–2 days old virgin males) which had been reared at controlled densities. For this, F0 females and males of each line were placed into small round plastic containers (diameter 10 cm; height 11.5 cm; with filter paper on the bottom and a lid with a thrips mesh-covered opening), containing one fully ripe lemon fruit each, to oviposit for 24 h after which the adults were removed (Fig. 1). An ad libitum amount of *Typha* sp. pollen was added to increase female fecundity (Varikou et al. 2009). Upon pupation of their offspring, pupae of each line were separated by sex and transferred to individual Petri dishes (diameter 60 mm; height 15 mm) with a moist filter paper. One day after adult emergence (F1), each virgin female was transferred to a Petri dish (diameter 35 mm, height 10 mm) together with ten virgin males (1–2 days old) and *Typha* sp. pollen. Once mating had started with one male, the copulation activity of individual pairs was monitored to ensure that mating was not disrupted. Then each individual pair was transferred onto one lemon fruit in a small round plastic container (Nguyen et al. 2017; Katlav et al. 2021a) and ad libitum *Typha* sp. pollen was added and replenished every second day. The pairs were removed after the mated females were allowed to lay eggs, first for 14 days in experiments 1 and 2, and then for a total of ten days in experiment 3 because we found that most females laid their eggs within the first ten days. Offspring were reared until adulthood (F2) for recording of the offspring number and sex ratio (Fig. 1).

### Effects of endosymbionts and temperature on offspring number and sex ratio

Upon emergence of all adults, number and sex ratio of offspring (F2) produced by each individual mother was recorded. As in other similar studies, any pair that had produced fewer than ten offspring was excluded (Snook et al. 2000). In our study this applied to only one U × U pair in experiment 2 and 3 each. Offspring sex ratio of each individual female was calculated as the percentage of male offspring in the brood. Consistent with a previous study (Katlav et al. 2021a), females showed a bimodal sex ratio pattern and were thus grouped into two categories depending on the percentage of male offspring: constrained females with 85–100% male offspring (M broods) and unconstrained females with 0–15% male offspring (F broods). Due to the bimodal nature of sex allocation in this species, a mixed effects logistic regression was used to analyse the effect of “infection status” and “temperature” (as two binary fixed factors; infected vs. uninfected; 20 vs. 25 °C) on the probability of constrained sex allocation. We incorporated “total offspring number” as covariate and “experiment number” as random factor. The mixed effects logistic regression analyses for sex ratio data were performed using a generalized linear mixed effects model (*glmer* function) with (“family = binary”; α = 0.05) in the “*lme4*” package in R version 3.5.1 (R Development Core Team 2015). We further assessed the role of endosymbiont and temperature on sex ratio for constrained and unconstrained females separately. According to a Shapiro–Wilk test the sex ratio data had a non-normal distribution; therefore, we used a Wilcoxon rank sum test in R to compare group means.

### Effect of temperature on CI

Previous experiments conducted at 20 °C demonstrated that *Cardinium* and *Wolbachia* coinfection caused complete CI of the FM-CI type in U × I_CW_ crosses of *P. kellyanus* (Nguyen et al. 2017). Besides compatible pairs (I_CW_ × I_CW_; U × U), experiment 3 also included incompatible pairs (U × I_CW_) at both 20 and 25 °C. This enabled us to assess whether the strength and type of CI changed with temperature. This was achieved by comparing the number of male vs. female offspring across the two temperatures. According to a Shapiro–Wilk test the normality assumption was not met for the CI data. Therefore, the CI dataset was processed using Aligned Rank Transform in the ARTool package (Wobbrock et al. 2011) in R to then perform a non-parametric ANOVA, as previously conducted for other CI studies (Nguyen et al. 2017), followed by pairwise comparisons with Tukey post hoc tests.

### Quantification of endosymbiont density

In another experiment endosymbiont densities were measured after rearing thrips for two consecutive generations (G1-G2) across four different temperature regimes: (1) both generations at 20 °C (20–20); (2) both generations at 25 °C (25–25); (3) G1 at 25 °C and G2 at 20 °C (25–20); (4) G1 at 25 °C and G2 at 30 °C (25–30) (see Supplementary Information; Table S1). From each treatment three to five newly emerged G2 adult females and males were sampled, except for the 25–30 treatment for which most G1 adult mothers died due to unknown reasons, and offspring (G2) were obtained from only three females of unknown mating status, and they only produced males. The G2 individuals from all treatments were subjected to DNA extraction and quantitative PCR with *gyrB* as a *Cardinium* gene, *coxA* and *fbpA* as two *Wolbachia* genes, and *Ef1a* as a host reference gene (for details about DNA extraction and quantitative PCR see Supplementary Information; Tables S2–S3; Nguyen et al. 2017). Relative symbiont densities were calculated following normalisation to the host gene using 2^−∆Cq^ (Schmittgen and Livak 2008). The effects of “thermal regime” and “sex” on endosymbiont densities were analysed as fixed factors using a general linear mixed effect model (*lmer* function) with the “*lme4*” and “*car*” packages in R.

## Results

### Effects of endosymbionts and temperature on constrained sex allocation

**Fig. 2:**
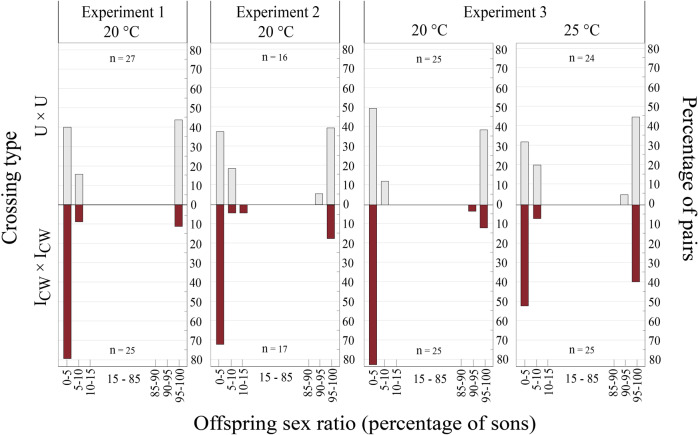
Frequency distribution of uninfected (U × U; top) and endosymbiont-infected (I_CW_ × I_CW_; bottom) pairs (% of pairs) producing particular offspring sex ratios (% of sons). The crossing experiments revealed bimodal sex ratio patterns, with some mated females producing male-biased (M) broods (constrained females) and other mated females producing female-biased (F) broods (unconstrained females). Experiments 1–2 evaluated the percentage of constrained and unconstrained mothers at 20 °C, and experiment 3 at 20 and 25 °C.

Mated *P. kellyanus* females displayed a bimodal sex ratio pattern, i.e., some mated females were not constrained and had F broods (0–15% males), whereas other mated females were constrained and had M broods (85–100% males) (Fig. 2). At 20 °C across all experiments, about 85% of 67 mated females with both endosymbionts (I_CW_) produced F broods with an average sex ratio of 1.7% males, while <58% of 66 mated females without endosymbionts (U) produced F broods with an average sex ratio of 4.5% males (Fig. 2). Therefore, mated U females were more likely constrained to M brood production (42% constrained females) than I_CW_ females (15% constrained females) (F_1,183_ = 9.89; *P* < 0.001). Moreover, in experiment 3 the proportion of females producing M broods was higher at 25 °C (38% of the 50 mated females) than at 20 °C (28% of the 133 mated females) (F_1,183_ = 4.28; *P* = 0.039). Because of a marginally significant interaction of temperature and endosymbiont infection status on the model (F_1,183_ = 3.61; *P* = 0.057), a step-down analysis of the endosymbiont effect on sex ratio was performed for each thermal treatment separately. This revealed that the effect of endosymbionts on M brood production was only detectable at 20 °C (F_1,133_ = 10.38; *P* < 0.001), whereas the M brood production was similar between mated I_CW_ and U females at 25 °C (F_1,50_ = 1.45; *P* = 0.21). Furthermore, our model did not detect an effect of constrained sex allocation on offspring number (F_1,183_ = 1.63; *P* = 0.09).

Further analyses of the offspring sex ratios of individual females showed that mated unconstrained I_CW_ females producing F broods had a lower percentage of male offspring (more female-biased) than mated unconstrained U females producing F broods at both 20 and 25 °C (Table 1). However, the percentage of male offspring of mated constrained females producing M broods did not differ between I_CW_ or U in both temperature treatments. Beyond this, for both mated I_CW_ and U females, temperature did not impact the sex ratio of F broods and M broods (Table 1).

**Table 1:** Comparison of sex ratios (as % males) in broods of unconstrained mated females (with F broods) and constrained mated females (M broods) of pairs with different endosymbiont infections (I_CW_ × I_CW_; U × U) at 20 and 25 °C (data of all three crossing experiments are pooled).

Temperature	Offspring type	Sex ratio median (1st–3rd quartile)		Wilcoxon rank sum test (effect of endosymbionts)
		I_CW_ × I_CW_	U × U	
20 °C	F broods	0 (0–3.03)	2.44 (0–4.35)	W = 792, ***P*** **=** **0.02**
	M broods	100 (100–100)	100 (100–100)	W = 127, *P* = 0.512
25 °C	F broods	0 (0–0)	5.56 (0–8.11)	W = 39, ***P*** **=** **0.002**
	M broods	100 (100–100)	100 (95.34–100)	W = 67.5, *P* = 0.057
Wilcoxon rank sum test (effect of temperature)	F broods	W = 565.6, *P* = 0.09	W = 172.5, *P* = 0.103	
	M broods	W = 40.50, *P* = 0.40	W = 199, *P* = 0.108	

Sex ratio is presented as median, with 1st and 3rd quartile values in parentheses. *P* values < 0.05 that are statistically significant are shown in bold.

### Effect of temperature on CI

**Fig. 3:**
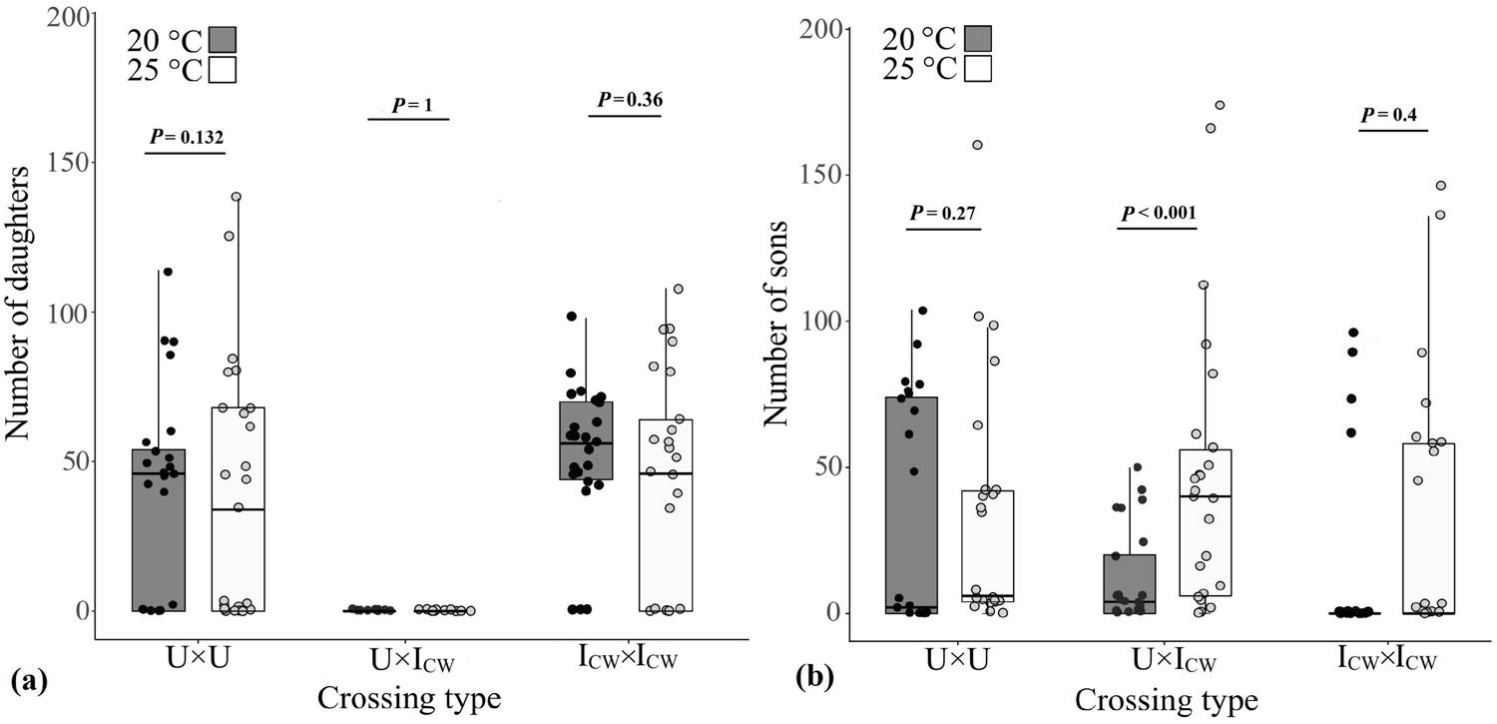
Adult offspring number of crossing types at different temperatures. Numbers of (**a**) adult female and (**b**) male offspring produced by different crossing types when reared at 20 or 25 °C (experiment 3). Box plots are represented with a jittered dot-plot overlay, with each dot representing the number of adult female or male offspring produced by individual pairs of compatible pairs of uninfected (U × U) or infected (I_CW_ × I_CW_) individuals as well as incompatible pairs (U × I_CW_) experiencing cytoplasmic incompatibility. Boxes include the median, the first and the third quartile range; whiskers represent the minimum and maximum range of the samples; outliers are shown as circles that did not fall within the bounds of whiskers.

All incompatible pairs (U × I_CW_) at 20 and 25 °C (experiment 3) resulted in male-only offspring, and, therefore the expression of CI was complete and independent of temperature (Fig. 3a; Table 2). The total number of offspring produced was significantly lower for incompatible pairs than for compatible pairs at both temperatures (Table 2), indicating that the coinfection of *Cardinium* and *Wolbachia* in males resulted in the mortality of fertilized eggs of uninfected females (FM-CI), as also observed in a previous study (Nguyen et al. 2017). However, the number of sons produced by U × I_CW_ pairs at 25 °C was nearly 40% higher than at 20 °C (F_1,50_ = 11.94; *P* < 0.001) (Fig. 3b; Table 2), suggesting a switch from FM-CI to a partial MD-CI at the higher temperature. Furthermore, at 25 °C, the U × I_CW_ pairs also produced more sons than the compatible I_CW_ × I_CW_ pairs further suggesting the expression of MD-CI (Table 2).

**Table 2:** Offspring number of *Pezothrips kellyanus* crosses (experiment 3) in response to the interactive effects of temperature and crossing type (endosymbiont infection).

Temperature	Crossing type ♀ × ♂	*N*	Male offspring Median (1st–3rd quartile)	Female offspring Median (1st–3rd quartile)	Total offspring Mean ± SE
20 °C	U × U	25	2 (0–74) a	46 (0–54) a	62 (50–80) a
	U × I_CW_	25	4 (0–20) ab	0 (0–0) b	4 (0–20) b
	I_CW_ × I_CW_	25	0 (0–0) b	56 (44–70) c	62 (48–72) a
			***P*** = **0.04**	***P*** < **0.001**	***P*** < **0.001**
25 °C	U × U	24	7 (4–42) ab	39 (2–62) a	49 (42–72) ab
	U × I_CW_	25	44 (7–57) a	0 (0–0) b	43 (6–56) b
	I_CW_ × I_CW_	25	1 (0–58) b	50 (0–80) a	62 (56–89) a
			***P*** = **0.035**	***P*** < **0.001**	***P*** = **0.039**
Full model	Temperature		*F*_(1, 149)_ = 7.77; ***P*** < **0.01**	*F*_(1, 149)_ = 0.23; *P* = 0.62	*F*_(1, 149)_ = 9.43; ***P*** = **0.002**
	Crossing type		*F*_(2, 149)_ = 0.94; *P* = 0.39	*F*_(2, 149)_ = 35.92; ***P*** < **0.001**	*F*_(2, 149)_ = 28.73; ***P*** < **0.001**
	Interaction		*F*_(2, 149)_ = 2.54; *P* = 0.08	*F*_(2, 149)_ = 0.38; *P* = 0.67	*F*_(2, 149)_ = 4.28; ***P*** = **0.045**

*N* is the number of pairs. Male, female and total offspring number are presented as median (1st and 3rd quartile). *P* values < 0.05 that are statistically significant are shown in bold. Pairwise comparisons were conducted among crossing types within each temperature treatment. Medians that are significantly different are followed by different letters.

### Effect of temperature on endosymbiont density

**Fig. 4:**
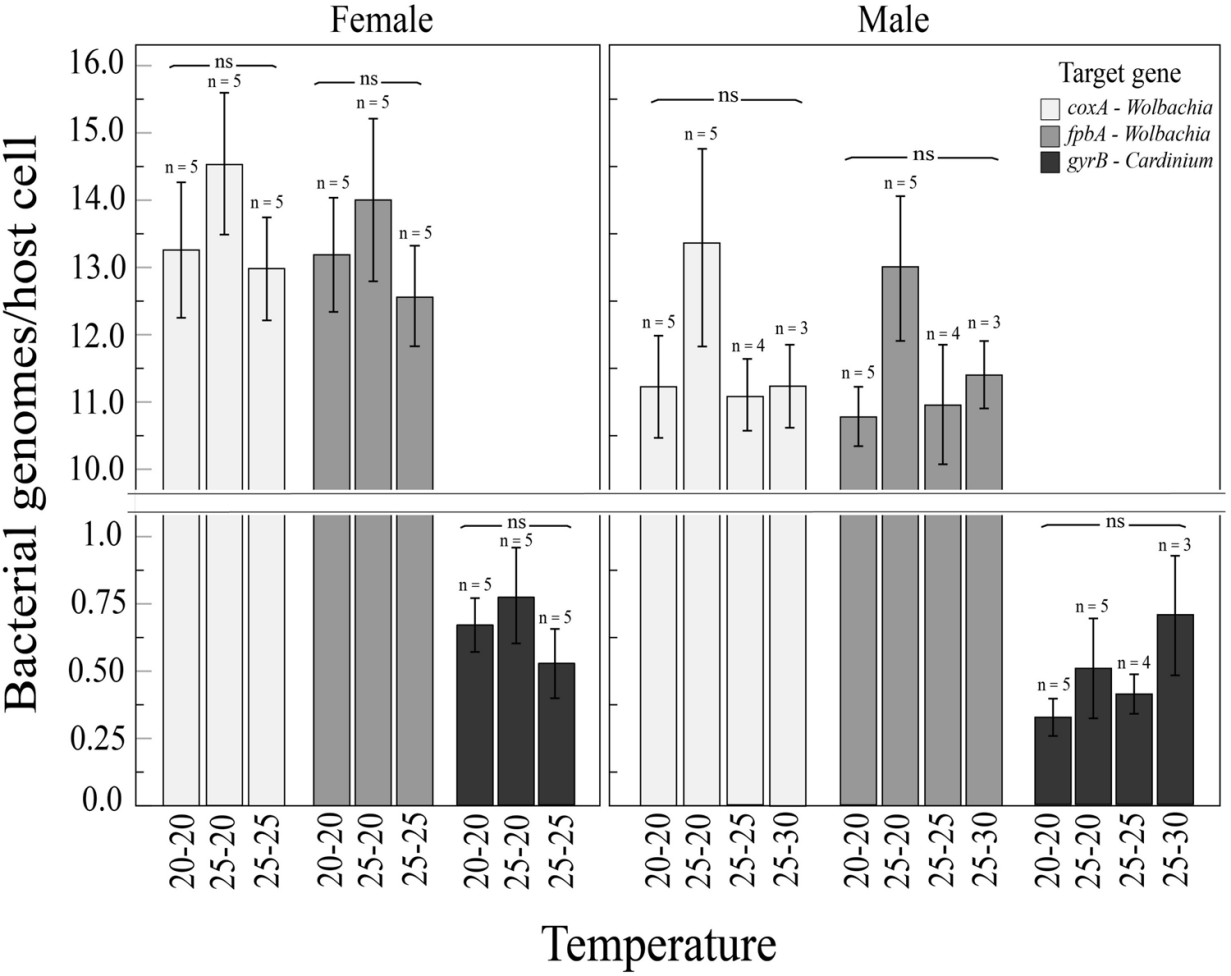
Comparison of *Cardinium* and *Wolbachia* density in I_CW_ females and males when reared at different temperatures across two successive generations (G1–G2). Mean (±SE) quantification cycle values (Cq) and individual data points of the single copy genes *coxA* and *fbpA* (for *Wolbachia* density) and *gyrB* (for *Cardinium* density) were normalized (2^−∆Cq^) to the host reference gene (*EF1a*). Error bars represent standard error and “ns” denotes non-significant difference of infections densities at *P* < 0.05 across temperature treatments, shown separately for each target gene in adult females and males.

Overall, *Wolbachia* densities were substantially higher (between 8 and 92-fold) than *Cardinium* densities. Furthermore, there was a strong effect of sex on *Wolbachia* densities with higher normalised Cq values for *fbpA* (F_1,32_ = 6.05; *P* = 0.021) and *coxA* (F_1,32_ = 4.91; *P* = 0.036) in females than in males. However, no effect of sex was detectable on *Cardinium* densities, with the analysis performed on *gyrB* Cq values (F_1,32_ = 3.08; *P* = 0.09). Furthermore, temperature did not significantly affect densities of *Wolbachia* (*fbpA*: F_3,32_ = 1.69; *P* = 0.19; *coxA*: F_3,32_ = 1.51; *P* = 0.21) or *Cardinium* (*gyrB*: F_3,32_ = 1.47; *P* = 0.24) (Fig. 4), and there was no interaction between sex and temperature for both endosymbionts densities (*fbpA*: F_2,32_ = 0.39; *P* = 0.68; *coxA*: F_2,32_ = 0.13; *P* = 0.87; *gyrB*: F_2,32_ = 0.37; *P* = 0.69).

## Discussion

We demonstrated that endosymbionts can moderate constrained sex allocation (M brood production) in a haplodiploid host species, and this occurred independently from any CI effects on sex allocation, and in a temperature-dependent way. We found that the proportion of *P. kellyanus* females mated with compatible males experiencing constrained sex allocation was diminished by the presence of both *Cardinium* and *Wolbachia* at 20 °C, and this endosymbiont effect was temperature-sensitive as it disappeared with the increase of temperature to 25 °C. These findings may explain previous studies which demonstrated that the population sex ratios of *P. kellyanus* became less female-biased when reared at higher temperatures in the laboratory (Varikou et al. 2012), and were seasonally fluctuating in field environments (Navarro-Campos et al. 2013).

Our finding of temperature-sensitive sex allocation is consistent with sex ratio patterns observed in several other haplodiploid taxa (Bondy and Hunter 2019). This has been recognized in some Hymenoptera species in which exposure to higher temperatures can cause increased maternal investment in male production (e.g., King 1987; Moiroux et al. 2014). Similar temperature-mediated sex ratio patterns were also found in whiteflies (Hemiptera), where this was suggested to be linked with a lower survival rate of females than males at higher temperatures (Cui et al. 2008). In a mealybug species (Hemiptera), higher male production observed at higher temperature (Nelson‐Rees 1960) was suggested to be associated with factors regulating paternal genome elimination or change in endosymbiont densities (Ross et al. 2010a). However, the potential role of endosymbionts in temperature-sensitive sex allocation patterns remained mostly unexplored in haplodiploids.

### Temperature effect on constrained sex allocation

Thermal stress, and in particular elevated temperature, can undermine the strength of endosymbiont-induced CI in incompatible crosses (Hoffmann et al. 1986; Corbin et al. 2017). In some hosts, weaker *Wolbachia*-induced CI was observed at higher temperatures and this was linked to the decline in endosymbiont density in male reproductive tissues (e.g., Breeuwer and Werren 1993; Ross et al. 2019). Nevertheless, a recent study of a *Cardinium*-infected parasitoid wasp species, *Encarsia suzannae*, showed that a temperature effect on CI was not associated with its effect on endosymbiont density but with the duration of the pupal stage when testes development occurs (Doremus et al. 2019). Furthermore, high temperature can result in CI becoming apparent in crosses that would otherwise be compatible. For example, *Wolbachia*-infected *Aedes aegypti* mosquito females which developed at high temperatures became partially incompatible with males reared at the same high temperature, probably because females lost their ability to rescue CI due to a lower *Wolbachia* density, while males largely retained the capacity to induce CI (Ross et al. 2019). In comparison, our study demonstrated for the incompatible crossing type U × I_CW_ stable levels of CI-induction across 20 and 25 °C (except for a shift from FM-CI to a partial MD-CI at the higher temperature), whereas, for the compatible (rescuing) crossing type I_CW_ × I_CW_ it was found that the frequency of females suffering from constrained sex allocation (and therefore producing only males) increased at the higher temperature. This could suggest temperature-sensitive changes in the rescue (but not the induction) of CI. However, we found that *Cardinium* and *Wolbachia* densities in both males and females remained steady across the examined temperature regimes. Therefore, increased constrained sex allocation seen in compatible crosses at the higher temperature may not be due to an overall decline in endosymbiont density in females. Nevertheless, CI may still arise in compatible crosses at higher temperatures due to a shift in endosymbiont localization patterns (Clark et al. 2003). If this was to apply then future experiments should test for any titre changes in reproductive tissues. However, we also note that there are examples of temperature-dependent *Wolbachia* titre changes that do not affect CI outcomes in hosts. For example, in the parasitoid *Leptopilina heterotoma* a temperature change from 14 to 25 °C did not influence the CI strength, despite a change in *Wolbachia* density (Mouton et al. 2006).

For *P. kellyanus* it has previously been shown that, at 20 °C, the coinfection of *Cardinium* and *Wolbachia* causes FM-CI, whereas *Cardinium* alone can induce moderate MD-CI; therefore, it was concluded that *Wolbachia* overrides the MD-CI of *Cardinium* (Nguyen et al. 2017). A dominance of FM-CI over MD-CI has also been shown for *L. heterotoma* coinfected with several *Wolbachia* strains with different CI types (Mouton et al. 2005). In our CI experiment, an increase from 20 to 25 °C caused increased male production. This may suggest a partial shift from FM-CI to MD-CI, possibly because the *Wolbachia* induced FM-CI function is more temperature-sensitive than the *Cardinium* MD-CI function.

We further hypothesize that the increased male production at the higher temperature may be due to an increase in constrained sex allocation. We have previously found that constrained sex allocation in *P. kellyanus* is linked with lower fertilization rates (Katlav et al. 2021a). Therefore, increased constrained sex allocation may prevent the expression of CI (which is only ever manifested in fertilized embryos of haplodiploid hosts). Future research should investigate whether the increased male production seen at higher temperatures is due to changes in CI rescue and/or whether endosymbionts affect fertilization. Furthermore, and in the context of CI in haplodiploids, perhaps the differences in CI types (FM-CI vs. MD-CI) are a manifestation of endosymbiont-dependent changes in constrained sex allocation in host species, and this needs further testing.

### Do endosymbionts moderate constrained sex allocation via metabolic provisioning?

A role of metabolic provisioning of the host by endosymbionts has been demonstrated in several empirical studies (Newton and Rice 2020; Currin-Ross et al. 2021). For example, *Wolbachia* can increase female fecundity in *Drosophila* flies by influencing iron homeostasis (Brownlie et al. 2009). In filarial nematodes, *Wolbachia* provisions the host with heme and riboflavin (Foster et al. 2005). Likewise, in a spider species, synthesis of fat and free amino acids has been shown to be improved by a *Wolbachia* and *Cardinium* coinfection (Li et al. 2020), and in bedbugs, *Wolbachia* plays a nutritional role in vitamin B synthesis (Hosokawa et al. 2010). Similarly, in whiteflies, the endosymbionts *Hamiltonella* and *Arsenophonus* contribute to the biosynthesis of B vitamins that facilitate oogenesis and higher fertilization rate (Wang et al. 2020). Furthermore, a positive role of *Wolbachia* in the fertilization rate of haplodiploids has previously been reported for the spider mite *T. urticae* (Vala et al. 2003) and the parasitoid wasp *Habrobracon hebetor* (Bagheri et al. 2021), yet the underlying physiological mechanisms are unknown.

Recently, it has been demonstrated that egg size plays a role in the fertilization success of haplodiploids, e.g., in *P. kellyanus* (Katlav et al. 2021b) and *T. urticae* (Macke et al. 2011), with higher fertilization rates for larger eggs. It has also been revealed that constrained sex allocation in *P. kellyanus* is associated with maternal condition (but not paternal fitness or failure in sperm transfer), because constrained females are smaller and produce smaller eggs than unconstrained females (Katlav et al. 2021a). Possibly, the higher F brood production in I_CW_ × I_CW_ than U × U is associated with a potential role of *Cardinium* and/or *Wolbachia* in metabolic provisioning. Therefore, we further hypothesize that endosymbionts may interact with the host’s egg size-mediated fertilization system, and this needs further investigation. Furthermore, a study on Lepidoptera has suggested that fertilization success increases with the number of micropyles on the egg (Iossa et al. 2016). Whether the egg size-mediated fertilization in haplodiploids is linked with a variation in the number and/or size of micropyles, and whether endosymbionts play a role in this, also still remains to be explored.

### Constrained sex allocation as an adaptive or non-adaptive response to endosymbiont infection

Several studies have shown that biased sex ratio patterns at the adult stage may be associated with sex-specific developmental mortality which consequently also results in a reduced adult offspring number (Werren and Charnov 1978; Nagelkerke and Hardy 1994). However, here we showed that endosymbiont effects on sex ratio patterns did not affect adult offspring numbers. Therefore, the increased proportion of M broods in the infected population at higher temperature was not associated with a higher developmental mortality of female offspring, but probably a shift in the host’s sex allocation (primary sex ratio). This temperature-dependent adjustment of sex allocation in response to endosymbiont infection is consistent with two alternative processes:

1. *Non-adaptive response:* under this scenario, constrained sex allocation is a consequence of physiological constraints preventing egg fertilization. In *P. kellyanus*, sex allocation of a female is associated with her condition. Some females fail to meet a required fitness threshold (body size) to facilitate fertilization success and, therefore, female production. Such fitness-dependant sex allocation has been revealed for a parasitic wasp species (Seidelmann et al. 2010). A growing number of studies have shown that elevated temperature can shorten developmental time by accelerating the larval metabolism (e.g., Murray et al. 2013; Zulkifli et al. 2018). This faster development can, consequently, result in smaller adults (Stearns 1992). Varikou et al. (2009) showed such a negative relationship between temperature and development time in *P. kellaynus*. Therefore, faster development of offspring at higher temperature could lead to smaller females which consequently fail to sufficiently provision eggs to ensure fertilization (Katlav et al. 2021a).
2. *Adaptive response:* under this scenario, constrained sex allocation may have evolved as an adaptive strategy of females in response to their energy budget that is affected by endosymbiont infection. Katlav et. al (2021b) found females developing from smaller eggs suffer a higher mortality rate than males. Therefore, this strategy may allow females to avoid fertilization of eggs that are not sufficiently provisioned, which would then lead to unsuccessful female development. Similar adaptive decisions have been observed in the oviposition behaviour of several parasitoid wasp species, where females can pause abdominal movements to ensure fertilization of the egg by a sperm cell (Martel and Boivin 2007; Moiroux et al. 2014).

### Host-endosymbiont conflict over constrained sex allocation

Maternally inherited endosymbionts may favour females to produce more daughters because males are a dead end for endosymbionts (Egas et al. 2002). However, for some females it can be beneficial to increase male production, especially when the population sex ratio is female-biased. Such host-endosymbiont conflicts over sex allocation have received substantial attention (Hurst 1992; Werren and Beukeboom 1998; Ross et al. 2010b). In *P. kellyanus*, there may be an evolutionary arms race between the host and the endosymbionts over sex allocation. Endosymbionts may select for increased F brood production (Werren 2011); conversely, the host may select for increased M brood production to avoid a highly female-biased population sex ratio (Godfray 1990) caused by endosymbionts, resulting in constrained sex allocation patterns. We demonstrated that constrained sex allocation is more intense in females of an uninfected population from which endosymbionts had been removed. Constrained sex allocation may have evolved as a female strategy to avoid cost of CI (especially FM-CI) by avoiding fertilisation of their eggs with incompatible sperm. A similar post-copulatory strategy for minimizing risk of CI has been shown for polyandry of *Drosophila* fruit flies in which multiple-mated females can potentially exploit the higher competitive ability of sperm of uninfected over infected males (Champion de De Crespigny et al. 2006; Price and Wedell 2008). However, the higher probability of constrained sex allocation in females without endosymbionts may result in a lower chance for an uninfected population to grow. This is consistent with the observation that uninfected *P. kellyanus* individuals are scarce in field populations (Nguyen et al. 2016), indicating that the endosymbionts have been able to spread to fixation not only due to CI, but perhaps also due to a reduced constrained sex allocation caused by one or both endosymbionts.

## Conclusions

In summary, our study demonstrated that the role of common endosymbionts such as *Wolbachia* and *Cardinium* in host reproduction is beyond the induction of commonly recognized reproductive manipulations (like CI manifested between infected and uninfected individuals) and may occur through changes in sex allocation in compatible crosses that can lead to increased female production. In haplodiploid populations, production of uninfected males as an outcome of CI can limit the invasion success of CI, as uninfected females can maintain their fitness by mating with these uninfected males (Vavre et al. 2000). We propose that the observed effect of endosymbionts on sex allocation may constitute a strategy to compensate for the lower efficiency of CI to drive endosymbiont invasion of haplodiploid host populations. On the other hand, the constrained sex allocation might be an outcome of the conflicts between the different optimal sex ratios aspired to by host and endosymbionts, with the host preferring more moderate population sex ratios than the female-biased sex ratios caused by endosymbionts. Given that *Cardinium* and *Wolbachia* may have different evolutionary trajectories and molecular mechanisms to affect hosts (Penz et al. 2012), future studies are yet to explore the net effect of each endosymbiont on constrained sex allocation in *P. kellyanus* separately. Finally, the temperature-dependent endosymbiont effect on sex ratio in *P. kellyanus* may provide insights into the evolution of temperature-dependent sex allocation mechanisms in many other arthropods.

## Supplementary information

Supplementary Information

## Data Availability

The data used in this manuscript are available on figshare repository: 10.6084/m9.figshare.16456023.
