# Supplementary material for: Endosymbionts moderate constrained sex allocation in a haplodiploid thrips species in a temperature-sensitive way
Source: Heredity (Edinb). 2022 Feb 3;128(3):169–77. doi: 10.1038/s41437-022-00505-5 (PMC8897473; doi:10.1038/s41437-022-00505-5)
Supplement: Supplementary file 1 — Supplementary Information [file 41437_2022_505_MOESM1_ESM.docx]

**SUPPLEMENTARY INFORMATION**

**1. Supplementary Methods: qPCR protocol for the quantification of the endosymbiont density under different temperature regimes**

Endosymbiont densities were measured after rearing thrips for two consecutive generations (G1-G2) across four different temperature regimes: (1) two generations at 20 °C (20-20); (2) two generations at 25 °C (25-25); (3) G1 at 25 °C and G2 at 20 °C (25-20); (4) G1 at 25 °C and G2 at 30 °C (25-30). A total of 32 male and female specimens (Supplementary Table S1) were subjected to DNA extraction using a PureGene kit (Qiagen) according to the manufacturer’s protocol. Briefly, individual thrips were crushed in 300 µL of cell lysis solution and incubated with 1.5 µL Proteinase K (20 mg/mL) at 55 °C overnight. Samples were treated with 1.5 µL RNaseA (Sigma) and incubated for 15 min at 37 °C prior to thorough mixing with 100 µL protein precipitation solution. Following centrifugation at 16,000 g for 3 min, the supernatant was isopropanol precipitated, the pellet washed in 70 % ethanol and resuspended in 100 µL Qiagen nuclease-free H_2_O.

Primers for qPCR were designed using PrimerQuest (IDT) to one host and three symbiont genes (Supplementary Table S2). *EF1a* was chosen as a single copy reference gene. *Wolbachia* was detected using the *coxA* and *fbpA* loci of the *Wolbachia* B-group strain found as a single infection in this thrips species (MLST allele *coxA* 14 and *fbpA* 14). *Cardinium* was detected by amplification of the gyrase (*gyrB*) gene. Primer pairs were chosen to amplify similar small sized amplicons to maximise PCR efficiency.

Efficiency of qPCR primer pairs was tested on amplicons generated with *Wolbachia* primers CoxF1-CoxR1 and FbpBspecF1-FbpBspecR1, *Cardinium* primers gyrBFor-gyrBRev and thrips primers EF1aF-EF1aR (Supplementary Table S3) on seven thrips individuals, samples 1, 6, 11, 16, 21, 26 and 32 (Supplementary Table S1). PCR products were electrophoresed on 1 % agarose, the seven individual amplicons for each primer set excised, combined and isolated using Wizard SV Gel and PCR Clean-up system Kit (Promega). Each template was serially diluted seven times (1 in 10 dilution) and subjected to triplicate qPCR, to generate primer efficiency curves using Rotor-gene 6000 software (version 2.2.3) (Supplementary Table S3). As the efficiency ranged from 0.92 to 0.97, the qPCR analysis was performed using the Delta Cq (quantification cycle) model without efficiency correction.

All triplicate qPCR reactions were assembled in 100-well rotor-discs using a CAS-1200 pipetting robot (Corbett Research). Reactions contained 5 µL SensiMix SYBR (Bioline, Sydney, Australia), 0.4 µM of each primer and 4.2 µL template (total 10 µL). Each DNA sample was first diluted 1/10 with nuclease-free H_2_O. The thermal cycling profile is described in Supplementary Table S2. Reference and target gene Cq values were calculated from the arithmetic average of the technical replicate Cq values at the same threshold level. Relative symbiont titres were calculated following normalisation to the host *EF1a* gene copy number using 2^-∆Cq^. For each haploid male sample, the Cq value for *EF1a* was reduced by 1 to make it equivalent to the diploid female.

**2.** **Supplementary Tables S1-S3**

**Table S1.** Specimens reared under different temperature regimes and used for qPCR of endosymbiont density

| **Specimen ID** | **Sex of specimens** | **Temperature regime and description** |
| --- | --- | --- |
|  | | |
| 1 | **Female** | **25 °C (G1) - 20 °C (G2)**    G1 (parents) were reared at 25 °C (from egg to pupal stage). As soon as adults emerged, G1 adults were allowed to mate and then kept at 20 °C for reproduction. G2 adults were collected 1-3 days after emergence. |
| 2 |  |  |
| 3 |  |  |
| 4 |  |  |
| 5 |  |  |
| 6 | **Male** |  |
| 7 |  |  |
| 8 |  |  |
| 9 |  |  |
| 10 |  |  |
| 11 | **Female** | **20 °C (G1) - 20 °C (G2)**    Both G1 and G2 were reared at 20 °C. G2 adults were collected 1-3 days after emergence. |
| 12 |  |  |
| 13 |  |  |
| 14 |  |  |
| 15 |  |  |
| 16 | **Male** |  |
| 17 |  |  |
| 18 |  |  |
| 19 |  |  |
| 20 |  |  |
| 21 | **Female** | **25 °C (G1) - 25 °C (G2)**  Both G1 and G2 were reared at 25 °C. G2 adults were collected 1-3 days after emergence. |
| 22 |  |  |
| 23 |  |  |
| 24 |  |  |
| 25 |  |  |
| 26 | **Male** |  |
| 27 |  |  |
| 28 |  |  |
| 29 |  |  |
| 30 | **Male** | **25 °C (G1) - 30 °C (G2)**  G1 (parents) were reared at 25 °C (from egg to pupal stage). Upon adult emergence, G1 adults were allowed to mate and then reared at 30 °C for reproduction. G2 adults were collected 1-3 days after emergence. This experiment did not work as most F0 adults died. Offspring (G2) were obtained from only three parents and they were all males. |
| 31 |  |  |
| 32 |  |  |

**Table S2.** Primer and qPCR thermocycling details for qPCR assays

| **Primer name** | **Primer sequence (5’-3’)** | **Target gene** | **Amplicon size** |
| --- | --- | --- | --- |
| Cox14F | GCCAGTATTTGGTTATATGGGAATG | ***coxA*** *Wolbachia* | 103 |
| Cox14R | CTCGCTAAGCCCAACAGTA |  |  |
| Fbp14F | GCTGTAGTGCTATGGTCTTATCC | ***fbpA*** *Wolbachia* | 103 |
| Fbp14R | CGAGCAAAGCCGCTATATGA |  |  |
| CardgyrF | CTGGAATTTGTAGCCCACTTAGA | ***gyrB*** *Cardinium* | 102 |
| CardgyrR | TCATGGCAACCTGTACCATTAC |  |  |
| EF1for | ATCAAGAACGTCTCCGTCAAG | ***Ef1a*** *Pezothrips* | 102 |
| EF1rev | GATGACCTGGGCAGTGAAG |  |  |
| **qPCR cycles** | 95 °C × 10 min |  |  |
|  | 95 °C × 20 s, 60 °C × 30 s, 72 °C × 45 s (× 45 cycles) |  |  |
|  | Melt: 50 °C to 98 °C; 1 °C increment/5s |  |  |

**Table S3.** Primer efficiency

| **Primers** | **R** | **R2** | **M** | **B** | **E** | **Threshold** |
| --- | --- | --- | --- | --- | --- | --- |
| **Cox14F-R** | 0.99842 | 0.99684 | -3.395 | -4.746 | 0.97 | 0.0555 |
| **Fbp14F-R** | 0.99933 | 0.99867 | -3.396 | -6.180 | 0.97 | 0.0555 |
| **CardgyrF-R** | 0.99714 | 0.99428 | -3.420 | -1.470 | 0.96 | 0.0555 |
| **EF1for-rev** | 0.99877 | 0.99754 | -3.521 | -0.212 | 0.92 | 0.0555 |

*dynamic tube, no slope correction

M = slope, B = Y-intercept, E = efficiency
